# Supplementary material for: The Small RNA Universe of Capitella teleta
Source: Front Mol Biosci. 2022 Feb 25;9:802814. doi: 10.3389/fmolb.2022.802814 (PMC8915122; doi:10.3389/fmolb.2022.802814)
Supplement: Supplementary file 1 [file DataSheet1.ZIP › Supplement/known/CAPTEscaffold_250_15832.pdf]

Provisional ID	: CAPTEscaffold_250_15832
Score total	: 160862.7
Score for star read(s)	: 3.9
Score for read counts	: 160853
Score for mfe	: 1.2
Score for randfold	: 1.6
Score for cons. seed	: 3
Total read count	: 315518
Mature read count	: 313751
Loop read count	: 0
Star read count	: 1767

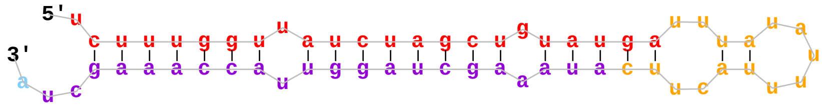

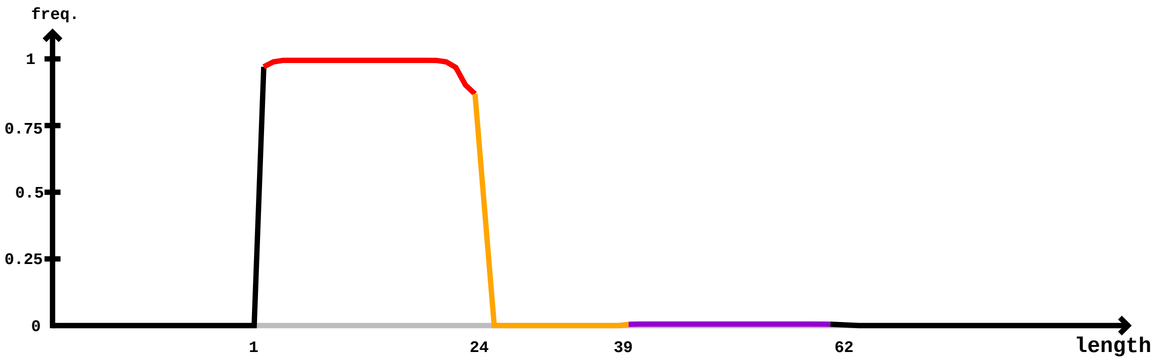

Mature **Star**

```
5'- gcaugauaaccccauuuuucucuuugguuaucuagcuguaugauuuauauuuuacuucauaaagcuagguuaccaaagcuaaaacaggggacaaaaucuaccgaguuaca  -3'   obs
    gcaugauaaccccauuuuucucuuugguuaucuagcuguaugauuuauauuuuacuucauaaagcuagguuaccaaagcuaaaacaggggacaaaaucuaccgaguuaca         exp
    gcaugauaaccccauuuuucucuuugguuaucuagcuguaugauuuauauuuuacuucauaaagcuagguuaccaaagcuaaaacaggggacaaaaucuaccgaguuaca         known
    .........((((..((((..(((((((.((((((((.(((((..((.....))..))))).)))))))).)))))))..))))..))))....................    reads   mm    sample

    ..................ucucCuugguuaucuagcuguauga...................................................................    1       1     seq
    ..................ucuUuuugguuaucuagcuguauga...................................................................    1       1     seq
    ...................cucuuugguuaucuagcuguauga...................................................................    1       0     seq
    ....................ucuuugguCaucuagcug........................................................................    1       1     seq
    ....................ucuuugguuaucuagcuA........................................................................    1       1     seq
    ....................ucuuugguuaucuagcuU........................................................................    2       1     seq
    ....................ucuuugguuaucuaUcug........................................................................    1       1     seq
    ....................ucuuuAguuaucuagcug........................................................................    1       1     seq
    ....................ucuuugguuaucuagcug........................................................................    99      0     seq
    ....................ucuuugguuaucuagcAgu.......................................................................    1       1     seq
    ....................ucuuugguuaucuGgcugu.......................................................................    7       1     seq
    ....................uAuuugguuaucuagcugu.......................................................................    2       1     seq
    ....................ucuAugguuaucuagcugu.......................................................................    1       1     seq
    ....................ucuuugguuaucuagcugA.......................................................................    7       1     seq
    ....................ucuuugguuaucuagUugu.......................................................................    3       1     seq
    ....................ucuuugguuUucuagcugu.......................................................................    1       1     seq
    ....................ucuuugguuaucuagAugu.......................................................................    1       1     seq
    ....................Acuuugguuaucuagcugu.......................................................................    3       1     seq
    ....................ucuuugguuGucuagcugu.......................................................................    1       1     seq
    ....................ucuuuggAuaucuagcugu.......................................................................    4       1     seq
    ....................ucuuugguuaucAagcugu.......................................................................    1       1     seq
    ....................ucuuugguuaucuagcugG.......................................................................    3       1     seq
    ....................ucuuugguuauAuagcugu.......................................................................    1       1     seq
    ....................ucuuAgguuaucuagcugu.......................................................................    1       1     seq
    ....................ucuuugguuaucuagcugC.......................................................................    1       1     seq
    ....................ucuuugguuaucuagcugu.......................................................................    1595    0     seq
    ....................ucuuugguuaucuagcuCu.......................................................................    1       1     seq
    ....................ucuAugguuaucuagcugua......................................................................    3       1     seq
    ....................ucAuugguuaucuagcugua......................................................................    7       1     seq
    ....................ucuuugguuaucuagcugAa......................................................................    2       1     seq
    ....................uAuuugguuaucuagcugua......................................................................    3       1     seq
```

```
                    Mature                                Star
gcaugauaaccccauuuuucucuuugguuaucuagcuguaugauuuauauuuuacuucauaaagcuagguuaccaaagcuaaaacaggggacaaaaucuaccgaguuaca
....................ucuuuggucaucuagcugua......................................................................   2      1   seq
....................ucuuugguuaucuagcuguU......................................................................   43     1   seq
....................ucuuuAguuaucuagcugua......................................................................   12     1   seq
....................uUuuugguuaucuagcugua......................................................................   3      1   seq
....................ucuuugguuaucuagcugGa......................................................................   1      1   seq
....................ucuuugguuaucuagcCgua......................................................................   1      1   seq
....................ucuuugguuaucAagcugua......................................................................   2      1   seq
....................ucuuugguuaucuaAcugua......................................................................   1      1   seq
....................ucuuugguuaucuagAugua......................................................................   4      1   seq
....................ucuuugguuaucuagcugCa......................................................................   1      1   seq
....................Acuuugguuaucuagcugua......................................................................   15     1   seq
....................ucuCgguuaucuagcugua......................................................................   2      1   seq
....................ucuuugCuuaucuagcugua......................................................................   1      1   seq
....................ucuuugguuauUuagcugua......................................................................   1      1   seq
....................Ncuuugguuaucuagcugua......................................................................   1      1   seq
....................ucuuugguuaucuagcuUua......................................................................   1      1   seq
....................ucuuugguuauAuagcugua......................................................................   2      1   seq
....................ucuuugguuaucuagUugua......................................................................   2      1   seq
....................ucuuugguuaucGagcugua......................................................................   1      1   seq
....................Gcuuugguuaucuagcugua......................................................................   5      1   seq
....................ucuuugguuaucuagcuguG......................................................................   11     1   seq
....................ucuuAgguuaucuagcugua......................................................................   4      1   seq
....................ucuuuggAuaucuagcugua......................................................................   3      1   seq
....................ucuuugguuaucuGgcugua......................................................................   12     1   seq
....................ucuuugguuGucuagcugua......................................................................   1      1   seq
....................ucuuugUuuaucuagcugua......................................................................   2      1   seq
....................ucuuuggCuaucuagcugua......................................................................   1      1   seq
....................ucuuugguuUucuagcugua......................................................................   1      1   seq
....................ucuuugguuaucuagcugua......................................................................   6392   0   seq
....................ucuuugguuaucuagcuAua......................................................................   1      1   seq
....................ucuuugguAaucuagcugua......................................................................   1      1   seq
....................ucuuugguuaucuagcCguau.....................................................................   2      1   seq
....................ucuuugguuaucuagUuguau.....................................................................   3      1   seq
....................ucuCgguuaucuagcuguau.....................................................................   1      1   seq
....................ucAuugguuaucuagcuguau.....................................................................   11     1   seq
....................ucuuugguuaucGagcuguau.....................................................................   2      1   seq
....................ucuuugguuUucuagcuguau.....................................................................   4      1   seq
....................Acuuugguuaucuagcuguau.....................................................................   45     1   seq
....................ucuuugguuaucuagcuguUu.....................................................................   2      1   seq
....................ucuuugguuaucuagcGguau.....................................................................   3      1   seq
....................ucuuugguuaucuaAcuguau.....................................................................   3      1   seq
....................ucuuAgguuaucuagcuguau.....................................................................   14     1   seq
....................ucuuugguuauAuagcuguau.....................................................................   6      1   seq
....................ucuuugguuaucuagAuguau.....................................................................   7      1   seq
....................uAuuugguuaucuagcuguau.....................................................................   5      1   seq
....................ucuuugCuuaucuagcuguau.....................................................................   2      1   seq
....................ucuAugguuaucuagcuguau.....................................................................   6      1   seq
....................ucuuugguuaAcuagcuguau.....................................................................   7      1   seq
....................ucuuugguuaucuagcuUuau.....................................................................   2      1   seq
....................ucuuCgguuaucuagcuguau.....................................................................   4      1   seq
....................ucuuuggCuaucuagcuguau.....................................................................   1      1   seq
....................ucuuugguuaucuagcuguaG.....................................................................   2      1   seq
....................uUuuugguuaucuagcuguau.....................................................................   5      1   seq
....................ucuuugguuaucuagcugGau.....................................................................   2      1   seq
....................uNuuugguuaucuagcuguau.....................................................................   3      1   seq
....................ucuuugguuaucuagcuguaA.....................................................................   126    1   seq
....................ucuuugguuaGcuagcuguau.....................................................................   1      1   seq
....................ucuuugguuaucCagcuguau.....................................................................   4      1   seq
....................ucuuugguuaucAagcuguau.....................................................................   11     1   seq
....................ucuuugguuaucuagcuguau.....................................................................   20375  0   seq
....................ucuuugAuuaucuagcuguau.....................................................................   4      1   seq
....................ucuuugUuuaucuagcuguau.....................................................................   5      1   seq
....................ucuuugguuauGuagcuguau.....................................................................   2      1   seq
....................ucuuuAguuaucuagcuguau.....................................................................   31     1   seq
....................Ccuuugguuaucuagcuguau.....................................................................   4      1   seq
....................ucuuuUguuaucuagcuguau.....................................................................   1      1   seq
....................ucuuugguuaCcuagcuguau.....................................................................   1      1   seq
....................ucuuugguuaucuagcAguau.....................................................................   1      1   seq
....................ucuuugguuaucuagcugCau.....................................................................   3      1   seq
....................ucuuugguAaucuagcuguau.....................................................................   2      1   seq
```

```
                    Mature                                    Star
gcaugauaaccccauuuuucucuuugguuaucuagcuguaugauuuauauuuuacuucauaaagcuagguuaccaaagcuaaaacaggggacaaaaucuaccgaguuaca
....................ucuuugguuaucuagcuguaC.....................................................................    7      1    seq
....................ucuuugguuGucuagcuguau.....................................................................    2      1    seq
....................Gcuuugguuaucuagcuguau.....................................................................    12     1    seq
....................Ncuuugguuaucuagcuguau.....................................................................    4      1    seq
....................ucuuugguuauUuagcuguau.....................................................................    2      1    seq
....................ucuuugguCaucuagcuguau.....................................................................    3      1    seq
....................ucuuGgguuaucuagcuguau.....................................................................    1      1    seq
....................ucuuugguuaucuagcugAau.....................................................................    5      1    seq
....................ucuuugguuaucuGgcuguau.....................................................................    25     1    seq
....................ucuuugguuaucuagcuguGu.....................................................................    5      1    seq
....................ucuuuggAuaucuagcuguau.....................................................................    12     1    seq
....................ucuuugguuaucuagcuAuau.....................................................................    1      1    seq
....................ucuuuCguuaucuagcuguau.....................................................................    2      1    seq
....................ucuuugguuaucuagcuguUug....................................................................    1      1    seq
....................ucuuuCguuaucuagcuguaug....................................................................    2      1    seq
....................ucuuugguuaucAagcuguaug....................................................................    8      1    seq
....................ucuuugguuaAcuagcuguaug....................................................................    2      1    seq
....................uGuuugguuaucuagcuguaug....................................................................    1      1    seq
....................uAuuugguuaucuagcuguaug....................................................................    2      1    seq
....................ucuuugguuaucuagGuguaug....................................................................    1      1    seq
....................ucuuugguuauUuagcuguaug....................................................................    3      1    seq
....................Ncuuugguuaucuagcuguaug....................................................................    4      1    seq
....................ucuuugUuuaucuagcuguaug....................................................................    2      1    seq
....................ucuuuAguuaucuagcuguaug....................................................................    11     1    seq
....................ucuuugguuaucuUgcuguaug....................................................................    1      1    seq
....................ucuuugguuaucuagAuguaug....................................................................    1      1    seq
....................ucuuugguuaucuagcuguauU....................................................................    311    1    seq
....................ucuuugguuaucuagcuguaAg....................................................................    3      1    seq
....................ucuuugguAaucuagcuguaug....................................................................    1      1    seq
....................ucuuugguuauAuagcuguaug....................................................................    3      1    seq
....................ucuuugguuaucuagcuguGug....................................................................    4      1    seq
....................ucuuugguuaucCagcuguaug....................................................................    3      1    seq
....................Acuuugguuaucuagcuguaug....................................................................    29     1    seq
....................ucuuugguuaucuGgcuguaug....................................................................    16     1    seq
....................ucuuugguuaucuagcuAuaug....................................................................    1      1    seq
....................ucuuugguuaCcuagcuguaug....................................................................    1      1    seq
....................ucuuugguuaucuagcuguaGg....................................................................    1      1    seq
....................ucuuugguuaucuagcAguaug....................................................................    3      1    seq
....................ucuuugguuaucuagUuguaug....................................................................    1      1    seq
....................ucuuugguuaucuagcuguaug....................................................................    9967   0    seq
....................ucCuugguuaucuagcuguaug....................................................................    3      1    seq
....................ucuAugguuaucuagcuguaug....................................................................    2      1    seq
....................ucuuugguuaucuagcugGaug....................................................................    1      1    seq
....................ucuuCgguuaucuagcuguaug....................................................................    1      1    seq
....................ucuuugguuaucuagcCguaug....................................................................    1      1    seq
....................ucuuuggAuaucuagcuguaug....................................................................    8      1    seq
....................Gcuuugguuaucuagcuguaug....................................................................    5      1    seq
....................ucuuugguuaucuagcuguaCg....................................................................    2      1    seq
....................ucuuugguuaucuagcuguauA....................................................................    77     1    seq
....................ucAuugguuaucuagcuguaug....................................................................    8      1    seq
....................ucuuugguCaucuagcuguaug....................................................................    2      1    seq
....................ucuuAgguuaucuagcuguaug....................................................................    4      1    seq
....................ucNuugguuaucuagcuguauga...................................................................    3      1    seq
....................ucuuugUuuaucuagcuguauga...................................................................    34     1    seq
....................ucuuugguuaucuaAcuguauga...................................................................    25     1    seq
....................ucuuugguuaucuagAuguauga...................................................................    38     1    seq
....................ucAuugguuaucuagcuguauga...................................................................    113    1    seq
....................uAuuugguuaucuagcuguauga...................................................................    76     1    seq
....................ucuuugguuaucGagcuguauga...................................................................    28     1    seq
....................Ncuuugguuaucuagcuguauga...................................................................    89     1    seq
....................Gcuuugguuaucuagcuguauga...................................................................    114    1    seq
....................ucuuugguuNucuagcuguauga...................................................................    1      1    seq
....................ucuuugguuaucuagcugAauga...................................................................    60     1    seq
....................ucuuugguuGucuagcuguauga...................................................................    37     1    seq
....................ucuuugguuaucuagcuguaugN...................................................................    3      1    seq
....................ucuuugguuauUuagcuguauga...................................................................    61     1    seq
....................ucuuugguuaucuagcuUuauga...................................................................    25     1    seq
....................ucuCugguuaucuagcuguauga...................................................................    23     1    seq
....................ucuuugguuauGuagcuguauga...................................................................    5      1    seq
....................ucuuuUguuaucuagcuguauga...................................................................    43     1    seq
```

```
                    Mature                           Star
gcaugauaaccccauuuuucucuuugguuaucuagcuguaugauuuauauuuuacuucauaaagcuagguuaccaaagcuaaaacaggggacaaaaucuaccgaguuaca
....................ucuuugguuaucCagcuguauga...................................................................	28	1	seq
....................ucuuugguuauAuagcuguauga...................................................................	97	1	seq
....................ucuuGgguuaucuagcuguauga...................................................................	5	1	seq
....................ucuuugguuaucuagcugCauga...................................................................	35	1	seq
....................ucuuugguuaucuagcuAuauga...................................................................	41	1	seq
....................ucuuugguuaucuagcuguauga...................................................................	259297	0	seq
....................ucuuCgguuaucuagcuguauga...................................................................	30	1	seq
....................ucuuuggCuaucuagcuguauga...................................................................	38	1	seq
....................ucuuugguuCucuagcuguauga...................................................................	1	1	seq
....................ucuuugguGaucuagcuguauga...................................................................	9	1	seq
....................uNuuugguuaucuagcuguauga...................................................................	9	1	seq
....................ucuuugguuaucuagcuguGuga...................................................................	254	1	seq
....................ucuuugguAaucuagcuguauga...................................................................	40	1	seq
....................ucuuuCguuaucuagcuguauga...................................................................	16	1	seq
....................ucuuugguuaucuagcAguauga...................................................................	50	1	seq
....................ucuuugguuaAcuagcuguauga...................................................................	93	1	seq
....................ucuuugguuaucuagcCguauga...................................................................	46	1	seq
....................ucuuugguuaucuagcuguaGga...................................................................	24	1	seq
....................ucuuugCuuaucuagcuguauga...................................................................	8	1	seq
....................ucuuugAuuaucuagcuguauga...................................................................	45	1	seq
....................ucuGggguuaucuagcuguauga...................................................................	4	1	seq
....................ucuuuggGuaucuagcuguauga...................................................................	14	1	seq
....................ucuuugguuaucuagcugGauga...................................................................	18	1	seq
....................ucuuugguuaucuaUcuguauga...................................................................	17	1	seq
....................ucuuugguuaucuagcuguaAga...................................................................	58	1	seq
....................ucuuugguuaucuagcuguauCa...................................................................	22	1	seq
....................ucuuuNguuaucuagcuguauga...................................................................	1	1	seq
....................ucuuugguuaucuaCcuguauga...................................................................	10	1	seq
....................ucuuugguuaucuagcuguaugG...................................................................	196	1	seq
....................uGuuugguuaucuagcuguauga...................................................................	5	1	seq
....................Acuuugguuaucuagcuguauga...................................................................	607	1	seq
....................ucuuugguuaucuagcuguaugU...................................................................	1060	1	seq
....................ucuuugguuaucuagcuguauAa...................................................................	328	1	seq
....................ucuuugguuaucuagcuCuauga...................................................................	13	1	seq
....................ucuAugguuaucuagcuguauga...................................................................	89	1	seq
....................ucuuugguuaucuagcuguaCga...................................................................	27	1	seq
....................ucuuugguuaucuagUuguauga...................................................................	18	1	seq
....................ucuuugguuaucuCgcuguauga...................................................................	3	1	seq
....................ucuuugguuaucuagcuguaugC...................................................................	33	1	seq
....................Ccuuugguuaucuagcuguauga...................................................................	35	1	seq
....................ucuuugguuaGcuagcuguauga...................................................................	10	1	seq
....................ucuuuAguuaucuagcuguauga...................................................................	400	1	seq
....................ucuuugguuaucuagcuguCuga...................................................................	1	1	seq
....................ucuuuggAuaucuagcuguauga...................................................................	175	1	seq
....................ucuuugguuaucuagcuguauUa...................................................................	23	1	seq
....................ucuuAgguuaucuagcuguauga...................................................................	129	1	seq
....................ucuuugguuaucuGgcuguauga...................................................................	195	1	seq
....................ucuuugguuaucuagcuguUuga...................................................................	27	1	seq
....................ucuuugguuaucuUgcuguauga...................................................................	19	1	seq
....................ucuuugguuUucuagcuguauga...................................................................	26	1	seq
....................ucCuugguuaucuagcuguauga...................................................................	53	1	seq
....................ucGuugguuaucuagcuguauga...................................................................	5	1	seq
....................ucuuugguCaucuagcuguauga...................................................................	44	1	seq
....................ucuuugguuaucAagcuguauga...................................................................	74	1	seq
....................ucuuugguuaucuagGuguauga...................................................................	13	1	seq
....................ucuuugguuaCcuagcuguauga...................................................................	24	1	seq
....................ucuuugguuaucuagcGguauga...................................................................	14	1	seq
....................uUuuugguuaucuagcuguauga...................................................................	78	1	seq
....................ucuuugguuaucuagcuguGugau..................................................................	2	1	seq
....................ucuuugguuaucuGgcuguaugau..................................................................	6	1	seq
....................ucuuugguuaucuagcuguaugaA..................................................................	631	1	seq
....................ucuuugguuaucuagcuguaugaC..................................................................	15	1	seq
....................ucuuugguuaucuagcuguaugUu..................................................................	72	1	seq
....................ucuuugguCaucuagcuguaugau..................................................................	1	1	seq
....................uAuuugguuaucuagcuguaugau..................................................................	1	1	seq
....................ucuuuggCuaucuagcuguaugau..................................................................	1	1	seq
....................ucuuugguuaucuagcuguaugau..................................................................	909	0	seq
....................Acuuugguuaucuagcuguaugau..................................................................	5	1	seq
....................ucuuugguuaucuagAuguaugau..................................................................	2	1	seq
....................ucuuugguuaucuagcuguauAau..................................................................	1	1	seq
```

```
                    Mature                        Star
gcaugauaaccccauuuuucucuuugguuaucuagcuguaugauuuauauuuuacuucauaaagcuagguuaccaaagcuaaaacaggggacaaaaucuaccgaguuaca
....................Gcuuugguuaucuagcuguaugau..................................................................	1	1	seq
....................ucuuugguuaucuagcuguauUau..................................................................	1	1	seq
....................ucuuugguuaucuagcuguaugaN..................................................................	1	1	seq
....................ucuuugguuaucuagcuguaugaG..................................................................	1	1	seq
....................ucuuugguuaucuagcuguaugauA.................................................................	5	1	seq
....................ucuuugguuaucuagcuguaugauC.................................................................	3	1	seq
....................ucuuugguuaucuagcuguaugaGu.................................................................	3	1	seq
....................ucuuugguuaucuagcuguaugauu.................................................................	67	0	seq
....................ucuuugguuaucuagcuguaugUuu.................................................................	4	1	seq
....................ucuuugguuaucuagcuguaugauG.................................................................	2	1	seq
....................ucuuugguuaucuagcuguaugaAu.................................................................	23	1	seq
....................ucuuugguuaucuagcuguaugauuu................................................................	2	0	seq
.....................cuuugguuaucuagcugu.......................................................................	1	0	seq
.....................cuuugguuaucuagcugua......................................................................	19	0	seq
.....................cuuugguuaucuagcuguau.....................................................................	13	0	seq
.....................cuuugguuaucuagcuAuaug....................................................................	1	1	seq
.....................cuuugguuaucuagcuguaug....................................................................	76	0	seq
.....................cuuugguuaucuagcuguauAa...................................................................	12	1	seq
.....................cuuugguuaucuagcuguauga...................................................................	5534	0	seq
.....................cuuugUuuaucuagcuguauga...................................................................	1	1	seq
.....................cuuugguuaucuaUcuguauga...................................................................	1	1	seq
.....................cuuuggAuaucuagcuguauga...................................................................	6	1	seq
.....................cuuugguuaucuagcuguauCa...................................................................	1	1	seq
.....................cuuugguuaucAagcuguauga...................................................................	4	1	seq
.....................cuuugguuaucuagcuguaGga...................................................................	1	1	seq
.....................cuuugguuaucuagUuguauga...................................................................	1	1	seq
.....................cuuugguuauUuagcuguauga...................................................................	1	1	seq
.....................Auuugguuaucuagcuguauga...................................................................	2	1	seq
.....................cuuugguuaucuGgcuguauga...................................................................	35	1	seq
.....................cuuAgguuaucuagcuguauga...................................................................	3	1	seq
.....................cuuugguuaucuagcuguauUa...................................................................	1	1	seq
.....................cuuugguuaucuagcuguaugG...................................................................	2	1	seq
.....................cuuugguuaucuagAuguauga...................................................................	2	1	seq
.....................cuAugguuaucuagcuguauga...................................................................	3	1	seq
.....................cuuugguuauAuagcuguauga...................................................................	2	1	seq
.....................cuuugguuUucuagcuguauga...................................................................	3	1	seq
.....................cuuUgguuaucuagcuguauga...................................................................	2	1	seq
.....................cuuugAuuaucuagcuguauga...................................................................	1	1	seq
.....................cAuugguuaucuagcuguauga...................................................................	2	1	seq
.....................cuuugguuaCcuagcuguauga...................................................................	1	1	seq
.....................cuuugguAaucuagcuguauga...................................................................	3	1	seq
.....................Uuuugguuaucuagcuguauga...................................................................	3	1	seq
.....................cuuugguuaucuagcAguauga...................................................................	1	1	seq
.....................cuuugguuaucuagcugAauga...................................................................	1	1	seq
.....................cuuugguuaucuagcugCauga...................................................................	1	1	seq
.....................cuuugguuaucuagcuguGuga...................................................................	1	1	seq
.....................cuuugguuaAcuagcuguauga...................................................................	1	1	seq
.....................cuuugguuaucuagcuguaugU...................................................................	12	1	seq
.....................cuuCgguuaucuagcuguauga...................................................................	1	1	seq
.....................cuuugguuaucuagcuguaAga...................................................................	2	1	seq
.....................cuuugguuaucuagcuCuauga...................................................................	1	1	seq
.....................cuuugguuaucuagcuguaCga...................................................................	2	1	seq
.....................cCuugguuaucuagcuguauga...................................................................	1	1	seq
.....................cuuugguuGucuagcuguauga...................................................................	1	1	seq
.....................cuuuAguuaucuagcuguauga...................................................................	7	1	seq
.....................cuuugguuaucuagcuguaugau..................................................................	16	0	seq
.....................cuuugguuaucuagcuguaugaA..................................................................	30	1	seq
.....................cuuugguuaucuagcuguaugaG..................................................................	2	1	seq
.....................cuuugguuaucuagcuguaugauu.................................................................	4	0	seq
......................uuugguuaucuagcugua......................................................................	19	0	seq
......................uuugguuaucuagcuguau.....................................................................	1	0	seq
......................uCugguuaucuagcuguau.....................................................................	1	1	seq
......................uCugguuaucuagcuguaug....................................................................	1	1	seq
......................uuugguuaucuagcuguaug....................................................................	35	0	seq
......................uuugguuaucuGgcuguauga...................................................................	24	1	seq
......................Auugguuaucuagcuguauga...................................................................	6	1	seq
......................uuugguuaucuagcuguaGga...................................................................	1	1	seq
......................uuugguuaucuagcuguaugG...................................................................	1	1	seq
......................uuugguuauAuagcuguauga...................................................................	2	1	seq
......................uuuAguuaucuagcuguauga...................................................................	1	1	seq
```

```
                    Mature                                 Star
gcaugauaaccccauuuuucucuuugguuaucuagcuguaugauuuauauuuuacuucauaaagcuagguuaccaaagcuaaaacaggggacaaaaucuaccgaguuaca
......................uuugguuUucuagcuguauga...................................................................    1      1      seq
......................uuuggAuaucuagcuguauga...................................................................    1      1      seq
......................Guugguuaucuagcuguauga...................................................................    1      1      seq
......................uuugguuaucuagcuguaugU...................................................................    4      1      seq
......................uuugguuaucuagcugCauga...................................................................    1      1      seq
......................uCugguuaucuagcuguauga...................................................................    5      1      seq
......................uuugguuaucuagcuguaAga...................................................................    1      1      seq
......................uuugguuaucuagcuguauga...................................................................    1607   0      seq
......................uuugguAaucuagcuguauga...................................................................    1      1      seq
......................uuGgguuaucuagcuguauga...................................................................    1      1      seq
......................Nuugguuaucuagcuguauga...................................................................    1      1      seq
......................Cuugguuaucuagcuguauga...................................................................    3      1      seq
......................uuugguuaucuagcuguaugaA..................................................................    18     1      seq
......................uuugguuaucuagcuguaugau..................................................................    17     0      seq
......................uuugguuaucuGgcuguaugau..................................................................    3      1      seq
......................uuugguuaucuagcuguaugaAu.................................................................    2      1      seq
......................uuugguuaucuagcuguaugauu.................................................................    12     0      seq
.......................uugguuaucuagcuguauga...................................................................    6      0      seq
.......................uuUguuaucuagcuguauga...................................................................    1      1      seq
.......................uugguuaucuagcuguaugau..................................................................    1      0      seq
.........................gguuaucuagcuguauga...................................................................    2      0      seq
..........................................................auaaagcuagguuaccaa..................................    1      0      seq
..........................................................auaaGgcuagguuaccaaag................................    6      1      seq
..........................................................auaaagcuagguuaccaaag................................    17     0      seq
..........................................................auaGagcuagguuaccaaagc...............................    1      1      seq
..........................................................auGaagcuagguuaccaaagc...............................    1      1      seq
..........................................................auaaagcuagguuaccaaagc...............................    47     0      seq
..........................................................auaaGgcuagguuaccaaagc...............................    24     1      seq
..........................................................auaaagcuGgguuaccaaagcu..............................    9      1      seq
..........................................................auaaagAuagguuaccaaagcu..............................    2      1      seq
..........................................................auaaagcuagguuaccaaagcG..............................    2      1      seq
..........................................................auaaaCcuagguuaccaaagcu..............................    1      1      seq
..........................................................aAaaagcuagguuaccaaagcu..............................    1      1      seq
..........................................................auaaagcuagguuaccaaagAu..............................    1      1      seq
..........................................................auaGagcuagguuaccaaagcu..............................    5      1      seq
..........................................................auGaagcuagguuaccaaagcu..............................    3      1      seq
..........................................................auaaagUuagguuaccaaagcu..............................    1      1      seq
..........................................................auaaGgcuagguuaccaaagcu..............................    54     1      seq
..........................................................auaaagcuagguuacUaaagcu..............................    1      1      seq
..........................................................auaaagcuagguuaccaaGgcu..............................    2      1      seq
..........................................................auaaagcuagguuaccaaagcu..............................    1292   0      seq
..........................................................auaaagcuaggAuaccaaagcu..............................    1      1      seq
..........................................................auaaagcuagguuaccaaagcua.............................    2      0      seq
...........................................................uaaagcuagguuaccaaag................................    33     0      seq
...........................................................uaaagcuagguCaccaaagc...............................    1      1      seq
...........................................................uaaagcuagguuaccaaagc...............................    10     0      seq
...........................................................uaGagcuagguuaccaaagc...............................    6      1      seq
...........................................................uaaagcuagguuaccaaagAu..............................    1      1      seq
...........................................................uaaagcuagguuaccaaagcu..............................    129    0      seq
...........................................................uaaagcGagguuaccaaagcu..............................    1      1      seq
...........................................................uaaGgcuagguuaccaaagcu..............................    8      1      seq
...........................................................uaaagcuagguuaccaaagcua.............................    53     0      seq
...........................................................uaaGgcuagguuaccaaagcua.............................    3      1      seq
...........................................................uaGagcuagguuaccaaagcua.............................    4      1      seq
...........................................................uaaagcuagguuaccaaagcuaU............................    1      1      seq
............................................................aaagcuagguuaccaaag................................    1      0      seq
............................................................aaagcuagguuaccaaagc...............................    13     0      seq
............................................................aaagcuagguuaccaaagcu..............................    20     0      seq
............................................................aaagcuagguuaccaaagcua.............................    9      0      seq
```
